# EHMTI-0195. Cortical changes in medication-overuse headache

**DOI:** 10.1186/1129-2377-15-S1-E30

**Published:** 2014-09-18

**Authors:** F Riederer, M Schaer, AR Gantenbein, R Luechinger, L Michels, S Kollias, PS Sandor

**Affiliations:** 1 2nd Neurological Department, Neurological Center Rosenhuegel, Vienna, Austria; 2 Stanford University Medical Center, Stanford Cognitive & Systems Neuroscience Laboratory, Stanford Palo Alto CA 94304, USA; 3 Neurology, Rehaclinic Bad Zurzach, Bad Zurzach, Switzerland; 4 Institute for Biomedical Engineering, Swiss Federal Institute of Technology, Zurich, Switzerland; 5 Institute of Neuroradiology, University Hospital Zurich, Zurich, Switzerland

## Introduction

In medication-overuse headache (MOH) pain modulation is probably dysfunctional at cortical and subcortical level, resulting in disequilibrium between pain inhibition and facilitation. Volumetric grey matter changes have been found in cortical regions, but also in the brainstem [2], the latter being reversible after successful detoxification [1].

## Aims

Surface-based morphometric analyses should complement volumetric findings, providing more specificity in the metric affected (thickness vs. gyrification). Whereas cortical thickness alterations probably rely on altered trajectories of cortical maturation or neurodegenerative processes, cortical folding (gyrification) abnormalities are thought to reflect early alterations to brain development.

## Methods

In the present study we investigated cortical thickness and gyrification in 29 patients with MOH according to International Headache Society criteria and 29 age- and gender matched controls, using FreeSurfer. Correction for multiple comparisons was performed.

## Results

In patients with MOH cortical thickness was decreased in the left middle frontal gyrus (rostral part) compared to controls, whereas local gyrification was increased in the right fusiform gyrus and adjacent temporal regions, as well as in the right occipital pole.

## Conclusions

Decreased cortical thickness in frontal regions corresponds to decreased grey matter volume in similar regions. Increased local gyrification in the right fusiform gyrus corresponds to increased grey matter volume in the previous volumetric study. Increased gyrification in occipital regions might be related to increased susceptibility for cortical spreading depression.
